# Physical activity awareness and its relationship with motivation among college students: development and validation of a Multidimensional Scale

**DOI:** 10.3389/fpubh.2026.1856461

**Published:** 2026-06-19

**Authors:** Rulan Shangguan, Jiajing Xie, Xiaofen Hamilton

**Affiliations:** 1 School of Physical Education, South China University of Technology, Guangzhou, China; 2 Department of Curriculum and Instruction, College of Education, The University of Texas at Austin, Austin, TX, United States

**Keywords:** college students, health behavior, mixed methods, physical activity awareness, psychometric validation, scale development

## Abstract

**Introduction:**

Physical activity (PA) levels remain insufficient among young adults, particularly college students, whose behavioral patterns often persist into adulthood. Although psychological factors are recognized as important determinants of PA behavior, limited attention has been paid to how individuals perceive and interpret their own physical activity and surrounding context. This study aimed to develop and validate a multidimensional scale measuring college students' perceived PA awareness.

**Methods:**

A sequential mixed-method design was employed. In the qualitative phase, focus group interviews were conducted to explore college students' understanding of PA awareness. Based on thematic analysis, four dimensions were identified: personal PA awareness, social support awareness, environmental awareness, and PA recommendation awareness. An initial item pool was then developed and administered to 994 undergraduate students from eight universities. Confirmatory factor analysis (CFA) was conducted to examine the factorial validity of the scale, and internal consistency reliability was assessed using Cronbach's alpha coefficients.

**Results:**

CFA supported a stable four-factor structure with acceptable model fit indices (RMSEA = 0.046, SRMR = 0.035, CFI = 0.968). The subscales demonstrated satisfactory internal consistency, with Cronbach's ? values ranging from 0.741 to 0.919. These findings provide empirical support for the reliability and construct validity of the proposed scale.

**Discussion:**

The findings suggest that PA awareness is a multidimensional psychological construct reflecting both individual perceptions and contextual influences. The developed scale may contribute to a better understanding of motivational processes underlying physical activity engagement among college students and provide a structured framework for future research on psychological determinants of PA behavior.

## 1 Introduction

Over the past few decades, the global prevalence of overweight and obesity has increased dramatically, posing serious challenges to public health (1). Recent global estimates indicate that more than one billion people worldwide are now living with obesity, highlighting the rapid growth of the epidemic over recent decades (2) reported that overweight and obesity increased by 27.5% among adults and 47.1% among children worldwide. This rapid rise has raised concerns that the long-standing trend of increasing life expectancy may be reversed (3). Consequently, promoting healthy lifestyles has become a critical priority for global health.

Regular participation in physical activity (PA) is widely recognized as an essential component of a healthy lifestyle and an effective strategy for preventing obesity and chronic diseases (4). Extensive evidence indicates that regular PA reduces the risk of cardiovascular disease, diabetes, hypertension, certain cancers, depression, and premature mortality (5). Despite these well-established benefits, physical inactivity remains highly prevalent. Studies suggest that adults spend more than half of their monitored time engaged in sedentary behaviors (6), and only a minority meet recommended aerobic and muscle-strengthening activity guidelines (7). These findings reveal a persistent gap between recommended activity levels and actual participation.

Among different population groups, college students represent an important target for PA promotion. The transition from adolescence to adulthood is a critical developmental stage during which long-term health behaviors are established. However, many college students adopt sedentary lifestyles due to academic demands, time constraints, and lifestyle changes (8). Physical inactivity during the college years may persist into adulthood, increasing long-term health risks (9).

Various interventions have been implemented to promote PA among college students, including conceptual physical education courses and campus-based wellness programs (10, 11). However, evidence suggests that the effects of many interventions are modest and often short-lived (12). These mixed outcomes indicate that current interventions may not sufficiently address the psychological mechanisms underlying PA behavior.

One potential mechanism influencing health behavior is awareness. Awareness refers to an individual's recognition and understanding of internal states and external environments related to behavior. In contrast to knowledge, which reflects factual understanding, awareness involves the perception and evaluation of one's own behaviors and surrounding contexts. Awareness is considered an important component of self-regulation because individuals must first recognize their current behavior and relevant standards before they can modify their actions.

Self-awareness theory (13) provides a useful framework for understanding this process. The theory suggests that individuals evaluate their behavior by comparing their current actions with internal standards or goals. When discrepancies between behavior and standards are recognized, individuals may attempt to adjust their behavior accordingly. In the context of PA, awareness of personal activity levels and awareness of recommended activity guidelines may motivate behavior change.

In addition to internal awareness, environmental factors also influence PA participation. The Social Ecological Model (SEM) emphasizes that health behaviors are shaped by multiple levels of influence, including individual, social, and environmental factors (14, 15). For college students, these factors may include peer support, campus culture, and the availability of recreational facilities.

Despite the theoretical importance of awareness in behavior regulation, empirical research examining PA awareness remains limited. Existing studies tend to examine cognitive or environmental determinants in isolation, and few studies have examined awareness as a multidimensional construct integrating both internal and contextual factors. Moreover, PA awareness is often conflated with related constructs such as physical activity knowledge, self-efficacy, and health literacy. However, these constructs differ conceptually. Knowledge refers to factual understanding of PA guidelines, self-efficacy reflects confidence in one's ability to perform PA, and health literacy emphasizes the ability to access and apply health information. In contrast, PA awareness involves the perception and evaluation of one's own behavior in relation to both internal standards and external contexts.

Therefore, the purpose of this study was to develop and validate a scale measuring self-perceived PA awareness among college students, grounded in self-awareness theory and the social ecological model. A sequential mixed-method design was employed. In the first phase, qualitative methods were used to explore how college students conceptualize PA awareness and to identify potential dimensions of the construct. In the second phase, a quantitative survey was conducted to examine the factorial structure, validity, and reliability of the developed scale.

## 2 Materials and methods

### 2.1 Research design

The present study adopted a sequential mixed-method design to explore and measure physical activity (PA) awareness among college students. Due to the limited existing research on PA awareness, qualitative methods were first employed to explore how college students perceive and conceptualize PA awareness. The findings from the qualitative phase informed the development of domains and items for a quantitative measurement instrument.

The research consisted of two sequential phases. In Study I, a phenomenographical approach using focus group interviews was employed to explore college students' understanding of PA awareness and to identify potential conceptual domains of the construct. The qualitative results were analyzed and synthesized to guide the development of scale items.

In Study II, a quantitative study was conducted to develop and validate an instrument measuring college students' PA awareness. The instrument was constructed based on the domains identified in Study I and relevant literature. Psychometric analyses, including factor analysis and reliability testing, were performed to evaluate the validity and reliability of the scale.

### 2.2 Study I: qualitative exploration of PA awareness

Study I employed a qualitative design to explore how college students perceive and interpret their physical activity (PA) awareness and to identify its underlying domains. A phenomenographic methodology was adopted to capture variations in students' conceptions of PA awareness, as this approach focuses on identifying qualitatively different ways in which individuals experience a phenomenon. This approach was appropriate because PA awareness reflects an internally constructed cognitive process that can be accessed through participants' descriptions of lived experience.

The analysis was guided by Marton and Booth's (52) awareness framework, which distinguishes between the internal horizon (individual interpretations and experiences) and the external horizon (contextual influences shaping those experiences). This framework enabled examination of both intrapersonal perceptions of PA and the broader social and environmental contexts in which these perceptions are formed.

Data were collected through five focus group interviews with 40 undergraduate students (21 male, 19 female) from a 4-year university. Each session lasted approximately 45 min and followed a semi-structured protocol exploring students' perceptions of PA, knowledge of PA recommendations, sources of PA-related information, and perceived facilitators and barriers to PA engagement. All discussions were audio-recorded and transcribed verbatim.

Data analysis followed an iterative phenomenographic process. Transcripts were read repeatedly to achieve immersion in the data. Meaningful segments reflecting students' conceptions of PA awareness were identified as meaning units and coded using inductive line-by-line open coding. All coding and theme development were conducted independently by two researchers. Initial coding discrepancies were resolved through consensus discussion, with inter-coder agreement calculated at 0.84, indicating strong reliability. Codes were continuously compared within and across transcripts using a constant comparative approach to identify similarities and variations in meaning.

Through iterative refinement, codes were grouped into preliminary categories representing qualitatively different ways of experiencing PA awareness. These categories were repeatedly reviewed, merged, and differentiated until stable and internally coherent structures were established. Higher-order themes were then abstracted from these categories, representing distinct dimensions of PA awareness.

Trustworthiness was ensured through multiple strategies. Analytic credibility was strengthened through peer debriefing within the research team throughout coding and theme development. Peer debriefing sessions were held biweekly throughout the analysis phase, with two researchers not involved in data collection reviewing coding decisions and challenging emerging interpretations. An audit trail was maintained to document coding decisions, category refinements, and analytic progress. Reflexive discussions were conducted to reduce potential researcher bias and ensure interpretations remained grounded in participants' accounts. A reflexive journal was maintained by the first author to document personal assumptions, methodological decisions, and analytical shifts, which was shared during team discussions to minimize bias. Analytic saturation was reached when no new conceptual categories emerged from the data. Member checking was not conducted due to the exploratory nature of the study and time constraints.

The analysis indicated that students' perceptions of PA were shaped by both personal interpretations and contextual influences. Participants commonly associated PA with structured exercise and reported limited systematic monitoring of their activity levels. Social relationships and environmental conditions were frequently identified as influencing PA engagement, whereas knowledge of PA recommendations was generally limited.

Four domains of PA awareness were identified: personal PA awareness, PA recommendation awareness, social support awareness, and environmental awareness. These domains were theoretically interpreted through integration of self-awareness theory and the social ecological model. Specifically, personal PA awareness and PA recommendation awareness reflect internal evaluative processes described in self-awareness theory, whereas social support and environmental awareness represent external contextual influences emphasized in the social ecological model. This structure suggests that PA awareness is a multidimensional construct integrating self-regulation and contextual perception, forming the conceptual basis for scale development in Study II.

### 2.3 Study II: scale development and validation

#### 2.3.1 Research AIMS and questions

This study aimed to develop an instrument that measures self-perceived PA awareness among college students guided by psychometric theories. The domains and items embedded in each domain were developed based on the findings in Study I and the previous literature. The following two research questions were addressed quantitatively.

1. What domains and items can adequately capture college students' PA self-awareness?

2. What are the validity and reliability of the scale designed to measure college students' PA awareness?

#### 2.3.2 Conceptual framework

Because limited research has systematically conceptualized PA awareness among college students, this study integrates self-awareness theory and the social ecological model to guide construct development. Self-awareness theory explains the internal evaluative processes underlying PA awareness, suggesting that individuals regulate behavior by comparing their current actions with internal standards. Accordingly, two domains are derived: personal PA awareness (perception of one's own activity level) and PA recommendation awareness (recognition of behavioral standards). The social ecological model extends this perspective by emphasizing external influences, including social and environmental contexts. Thus, two additional domains are conceptualized: social support awareness and environmental awareness. Together, these four domains represent an integrated structure of PA awareness, combining internal self-regulation (SAT) and external contextual influences (SEM).

#### 2.3.3 Self-awareness theory

**Figure 1:**
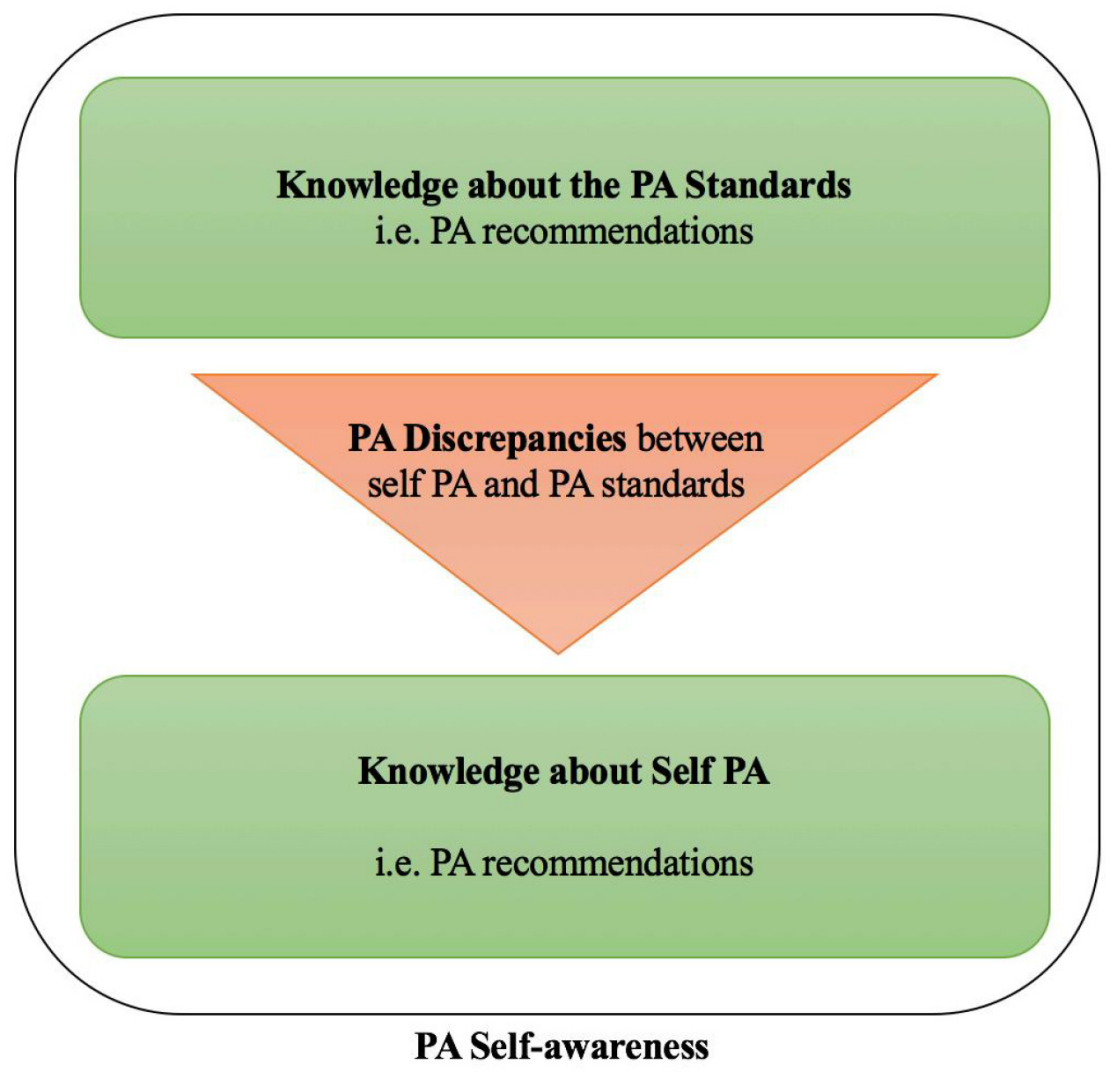
A conceptual framework of PA awareness based on self-awareness theory

According to Duval and Wicklund (13), when an individual focuses attention on the self and compare the self with standards that specify how one should think, feel or behave, referred as self-evaluation, the comparing process may lead to behavior change as an effort to reduce the discrepancies between their actions and ideals. Two key components are involved in the self-awareness process: knowledge about the standards and knowledge about self. Therefore, a framework based on self-awareness theory is used for constructing a PA self-awareness questionnaire to measure students' knowledge of PA standards and knowledge of personal PA levels in Figure 1.

#### 2.3.4 Social ecological model

**Figure 2:**
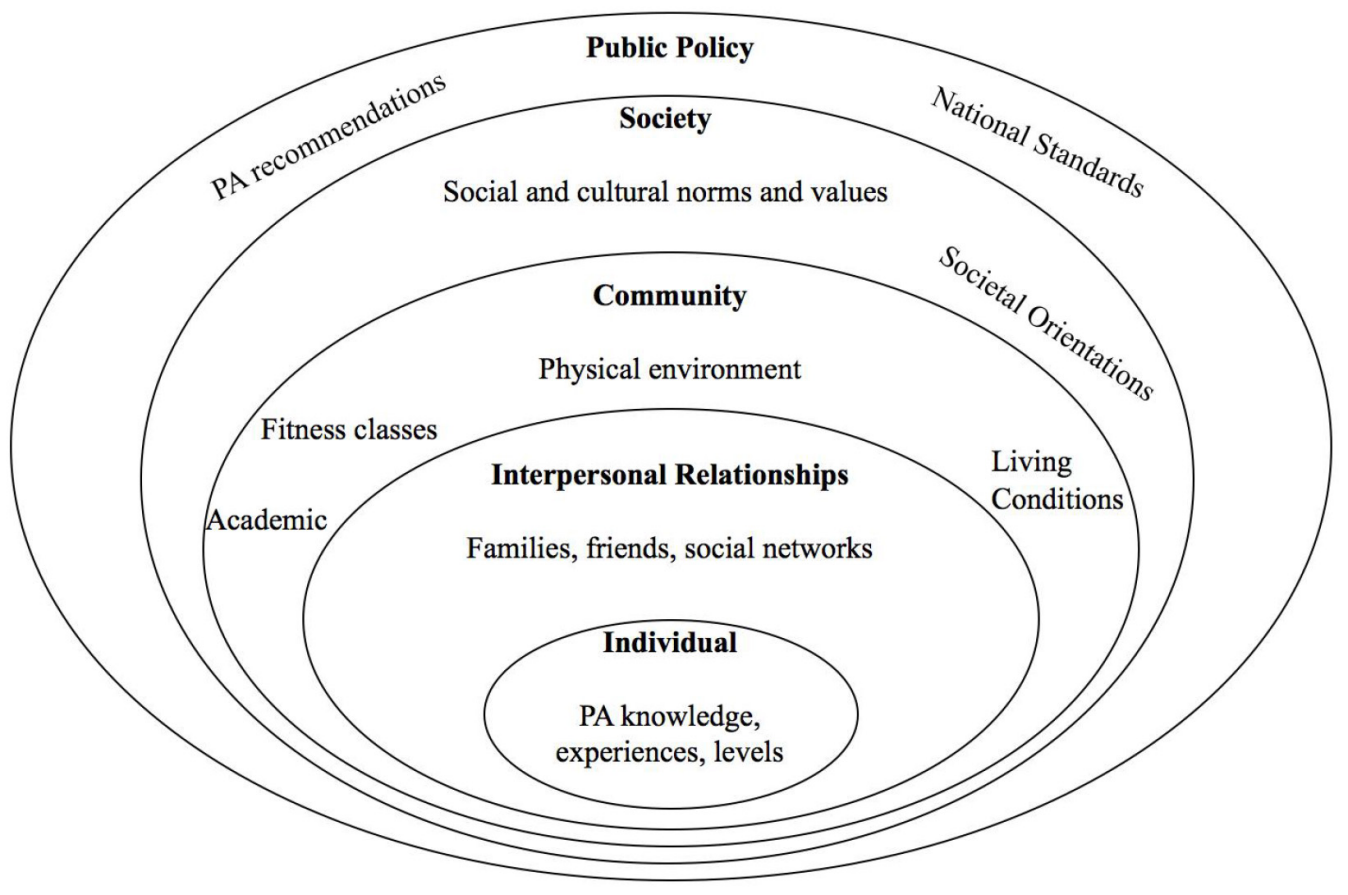
Adopted framework of college student PA awareness structure.

The social ecological model (SEM) illustrates the multifaceted and interactive effects of individual and environmental factors that determine behaviors (14). The SEM consists of multiple levels of factors that may alter one's behavior (16). According to the structure of awareness framework, awareness is made up of three overlapping aspects that could be divided into internal and external horizons. While self-awareness theory focuses on self as the object, the SEM explicitly pays attention to the self-environment relations that exist across different dimensions of the external horizon, including physical, social and cultural environment, to describe the reciprocal and dynamic relationships between self (the thematic field) and environment (the margin) (17). Categories derived from Study I were compared with the proposed models to define PA awareness domains, in which items measuring awareness were generated. Figure 2 demonstrated how PA awareness is interactively measured in the instrument as a combination of SAT and SEM.

## 3 Methodology

### 3.1 Research design

The purpose of this study was to determine whether the coverage of the instrument was appropriate and sufficient to measure PA awareness among college students. Initial items were written and firstly revised upon experts' feedback. Multiple phases were employed to achieve the goal of the study. The first phase focused on item construction to generate an initial version of questionnaire that assesses awareness of PA on four levels included in the SEM. The second phase involved the content validity study and the final phase was devoted to the test of the construct validity and reliability of PA awareness scale using a sample of college student for whom it is designed for.

### 3.2 Phase I: domain and item generation

Phase I aimed to develop a pool of items based upon the interview results of the previous study, and explore the item-domain relationships in the proposed PA awareness structure. Items were first drafted and discussed within a cohort of graduate students for feedback and comments, then reviewed by a professional writer for wording suggestions. The initial pool included items in three domains: (a) internal PA awareness consisted of a list of items regarding self-perceived levels of PA in three types (i.e. vigorous PA, moderate PA, and muscle strengthening PA) as well as respective personal goals, which were in line with the PA recommendation for adults by WHO; (b) external PA awareness included items related to perceived PA knowledge (i.e. PA recommendations), perceived social influence, and perceived physical environment especially related to the college setting that were found to be highly important from focus group results as well as previous research; (c) the interaction domain included items that focus on the extent to which individuals' PA behavioral decisions were influenced by aforementioned external factors (i.e. social and physical environment). Reverse worded items were used to ensure valid measures. Because PA awareness are conceptualized as propositions that one thinks to be true, all items were measured on a 7-point Likert scale, ranging from “strongly disagree” (i.e., scoring 1) to “strongly agree” (i.e., scoring 7) in order to generate variations needed for measuring latent variables (18).

### 3.3 Phase II: content validity

Phase II aimed to evaluate the content validity of the domains and items. Content validity refers to the extent to which the content of an instrument represents the content of an attribute it aims to measure. Experts in the field of interest are ideal judges who are often invited to critique the appropriateness of the instrument content, including professors, researchers and graduate students who have the expertise in the measured content area. The inter-item agreement among the experts is the evidence for content validity (18).

### 3.4 Procedure

Purposive sampling was used to recruit participants for the content validity study. A panel of experts (*N* = 10) in PA, fitness, health and measurement participated in the study. An online Qualtrics survey link of the instrument was sent to the experts by email, with the definition of PA awareness, and the definitions of the domains and subdomains. The experts were asked to evaluate the appropriateness of the domain and subdomains, the association between domain and subdomains, and the relevancy of items to the subdomains. In addition, the experts provided comments and suggestions concerning the domains and items, which were later incorporated in domain and item revisions.

## 4 Results

The experts' agreement on “internal awareness” domain was 80%, and the agreement on “external awareness” domain was 100%. However, only 70% agreement was achieved for the “interaction” domain, which consisted of items that were paralleled to the external to PA behaviors. Because of the low expert agreement and the experts' comments, the “interaction domain” and associated items were deleted. The scale consisted of 30 items after the expert review. Eliminating those items not only simplified the structure of the subdomains, but also shortened the survey and made it more time-economic for participants to complete.

### 4.1 Phase III: pilot testing

After removing irrelevant and unfit items suggested by the experts in the above content validity study, remaining items were randomly listed in the survey without the domain identification. The draft version was then sent to 50 college students for pilot testing, aiming to gather users' feedback regarding their comprehension of the item content, comfortableness to use the online format (i.e. web-based and smartphone based), as well as general concerns and questions. Wording and format were slightly revised based upon students' feedback from the pilot testing. Their input generated useful insights for the wording, design and distribution of the survey. For example, the online format included web-based and smartphone-based version, which presented different layout and flow in taking the entire survey. For smartphone users, the survey was tested multiple times using different operating systems to ensure ease in filling out each question.

### 4.2 Phase IV: field testing

The final version of the scale consisting of 30 items with two domains (four subdomains as indicated in the conceptual framework) was tested in 8 universities, with a total number of 1,122 undergraduate students participating in the study, among which 1,045 provided complete data, and 994 cases were reained after data screening for further analysis.

A set of questions regarding participants' demographic characteristics was included at the end of the survey, including age, gender, ethnicity, class standing, major, and living status. After data screening, a total of 994 valid responses were retained for analysis. The mean age of participants was 19.66 ± 3.66 years. The sample included a higher proportion of female students (60.2%) compared to males. Participants represented diverse ethnic backgrounds, including Caucasian (33.1%), Hispanic (31.3%), Asian (17.5%), and African American (12.1%) students. In terms of academic level, freshmen accounted for 34.2%, with other class standings distributed relatively evenly.

Participants were recruited from a wide range of academic majors, including Physical Education/Kinesiology (16.2%), Health-related majors (non-Physical Education/Kinesiology) (15.4%), STEM (24.0%), Liberal Arts (19.1%), Business (8.4%), Other majors (11.2%), and Undeclared students (3.2%). Regarding body mass index classification, most participants were within the normal weight range (60.9%), while 22.7% were overweight and 7.5% were obese. Detailed demographic characteristics are presented in Table 1.

**Table 1:** Participant demographic characteristics (study II).

Category	Subcategory	*N*	%
Sociodemographic characteristics			
Gender	Male	377	37.9%
	Female	599	60.2%
	Missing	18	18.1%
Ethnicity	African American	120	12.1%
	Asian	174	17.5%
	Caucasian	329	33.1%
	Hispanic/Latino	311	31.3%
	Native American	5	0.5%
	Other	37	3.7%
	Missing	18	18.11%
Class Standing	Freshmen	340	34.2%
	Sophomore	206	20.7%
	Junior	218	21.9%
	Senior	194	19.5%
	Other	15	1.5%
	Missing	21	2.1%
Health-related characteristics			
Major	Physical education/kinesiology	161	16.2%
	Health-related	153	15.4%
	Liberal arts	190	19.1%
	Business	83	8.4%
	STEM	239	24.0%
	Other	111	11.2%
	Undeclared	32	3.2%
	Missing	25	2.5%
SES	< $20,000	173	17.4%
	$20,000–49,999	160	16.1%
	$50,000–99,999	147	14.8%
	$100,000–199,999	163	16.4%
	>$200,000	74	7.4%
	Not sure	256	25.8%
	Missing	21	2.1%
BMI	Underweight	50	5.0%
	Normal	605	60.9%
	Overweight	226	22.7%
	Obese	75	7.5%
	Missing	38	3.8%
Living Status	On campus	308	31.0%
	Off campus	664	66.8%
	Missing	22	2.2%

### 4.3 Procedure

The recruitment of the participants took two forms: the researcher either contacted university class instructors to for their permissions to collect survey data in their class, or randomly distributed the survey to individual students around different campus areas, such as library, cafeteria, and other open study spaces. In addition, the survey data were collected in both online and paper versions. Participants recruited from existing classes were offered the options to respond either through a secured web-link to the questionnaire, or a paper format. Meanwhile, participants recruited individually from campuses were provided paper and pens to complete the survey on site. A consent page was provided at the beginning of the survey. All respondents received the items in the same order. The estimated time to complete the questionnaire was about 15 min to reduce the likelihood of disengaging respondents (19).

### 4.4 Data analysis

Data screening was conducted to first eliminate cases with more than 50% missing values, and then outliers to ensure normality. A total number of 994 cases remained for construct validity and internal consistency. Correlations of the 30 items were analyzed first in SPSS V21 to identify highly correlated items, followed by a confirmatory factor analysis (CFA) using SPSS Amos 21.0 to test construct validity. The remaining items after CFA were then analyzed for internal consistency using Cronbach's alpha in SPSS V21.

### 4.5 Construct validity

Construct validity examines the extent to which the relationships among items in the instrument are consistent with the theory and concepts (20). Factor analysis is appropriate for evaluating construct validity by revealing the constructs underlying item responses and determining item retention (21).

A CFA was used to test whether the hypothesized theoretical structure fit the observed data. When the initial model demonstrated inadequate fit, model respecification was conducted in an iterative manner using both statistical evidence (e.g., item diagnostics and modification indices) and theoretical considerations. Item retention decisions were based on cross-loadings, low standardized factor loadings, and conceptual inconsistency with the intended constructs. Items meeting these criteria were removed sequentially to improve factorial clarity. Modification indices were used as a supplementary diagnostic tool to identify areas of localized strain in the model. However, any modifications involving correlated errors or additional structural paths were retained only when they were theoretically defensible and consistent with physical activity awareness theory and prior empirical findings. Each modification was implemented stepwise, with model fit reassessed after each adjustment to ensure stability and interpretability of the model. The overall respecification process aimed to maintain a balance between statistical model fit and theoretical validity. Model fit was evaluated using multiple indices, including SRMR, RMSEA, GFI, NFI, and CFI. Recommended thresholds indicate acceptable fit when SRMR ≤ 0.08, RMSEA ≤ 0.06, GFI ≥ 0.90, NFI ≥ 0.95, and CFI ≥ 0.95 (53).

### 4.6 Internal consistency

Cronbach's alpha was used to measure the interrelatedness among items of the awareness scale to determine how well the items measure the same construct (22). Cronbach's alpha values ≥9 indicates excellent internal consistency, values ≥80 are considered to be good, and values ≥7 are considered to be moderate (22). Cronbach's alpha for both the total scale and subscales were calculated with values ≥70 to be acceptable (23). Pearson correlations for item-item, item-subscale, item-scale, subscale-subscale and subscale-scale were also calculated. Acceptable criterion for determining items to be kept are as following: (a) item-item correlations 0.30–0.70; (b) item-subscale correlations ≥50; (c) item-scale correlations ≥0.40; (d) subscale-subscale correlations 0.40–0.65; and (e) subscale-scale correlation 0.55–0.80 (22, 23).

## 5 Results

### 5.1 Confirmatory factor analysis results

**Figure 3:**
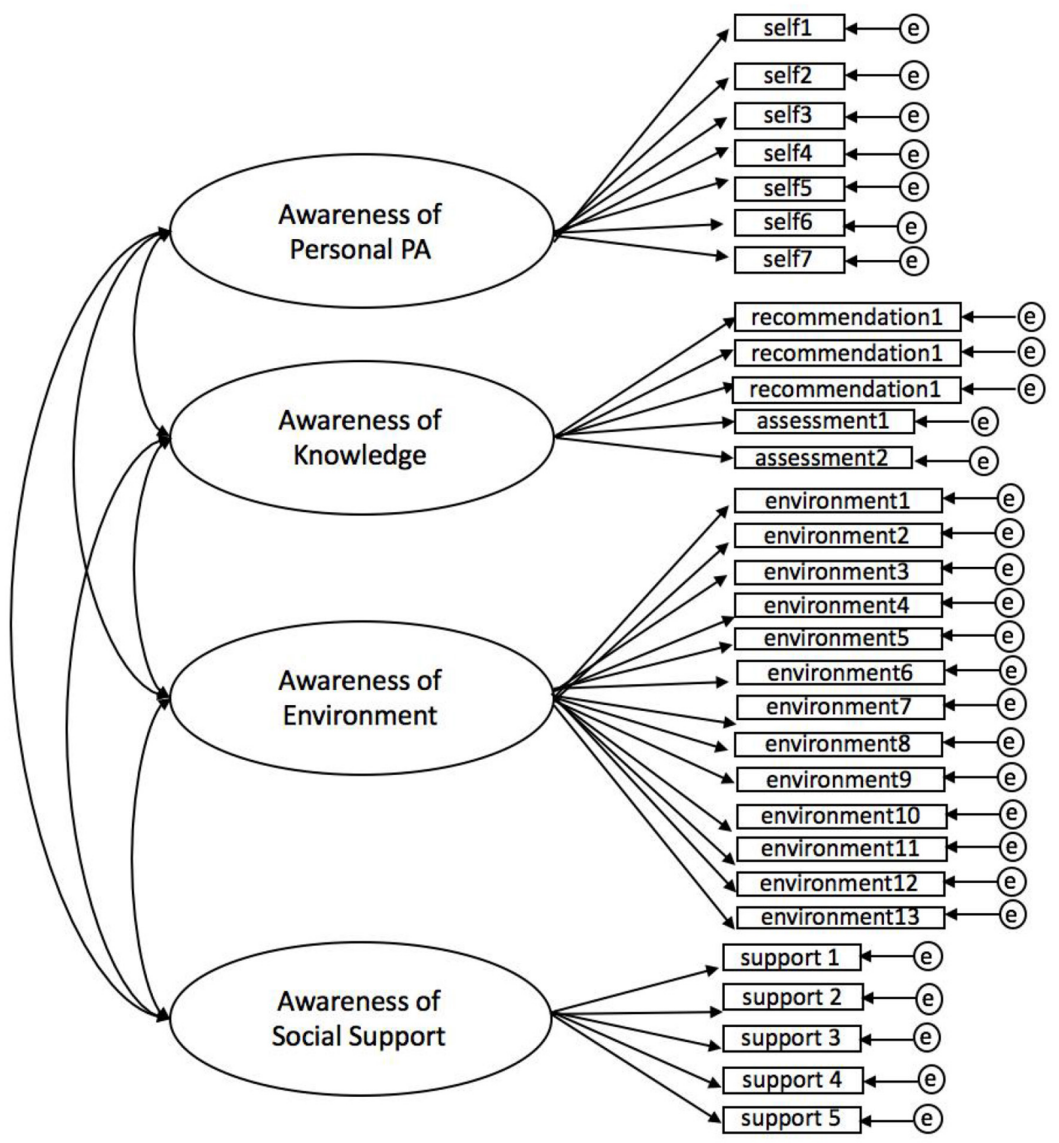
Structural diagram for the proposed model of physical activity awareness.

**Figure 4:**
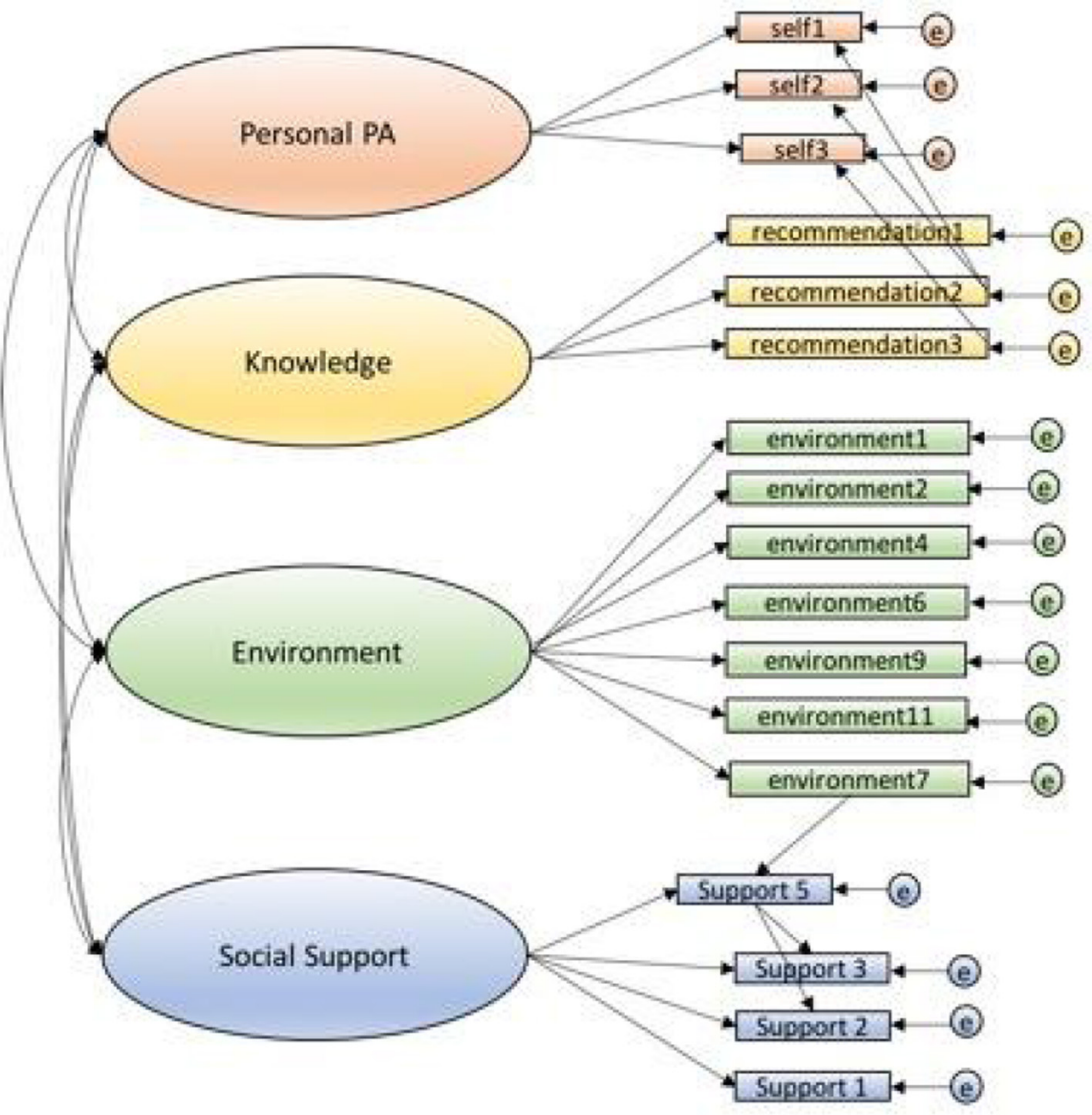
Structural diagram for the re-specified model of physical activity awareness.

A CFA (*N* = 994) was conducted to examine the factor structure proposed in the conceptual framework. The initial model demonstrated inadequate fit, as several fit indices did not meet recommended thresholds (see Figure 3). Therefore, model respecification was undertaken. Model respecification was guided by statistical diagnostics (i.e., modification indices and standardized factor loadings) and theoretical considerations. Item retention decisions were based on cross-loadings, low factor loadings, and conceptual inconsistency with the intended constructs. Based on these criteria, 13 items were removed. Four items showed substantial cross-loadings across factors, while nine items demonstrated conceptual redundancy with other indicators. These included items related to physical activity self-estimation methods, campus-wide health-related events, sedentary behavior estimation, personal goal setting, support-seeking behaviors, and environmental resources. In addition, six structural paths were added based on modification indices; however, these modifications were only retained when theoretically justified. These paths reflected conceptual linkages between instructional exposure and perceived support availability, as well as relationships between perceived knowledge of physical activity guidelines and self-reported behavioral estimates. All modifications were conducted iteratively, with model fit reassessed after each adjustment to ensure stability and theoretical interpretability. The final re-specified model (17 items) demonstrated acceptable to excellent fit (SRMR = 0.035, RMSEA = 0.046, GFI = 0.946, NFI = 0.954, CFI = 0.968), indicating improved alignment between the four-factor structure and the observed data (see Figure 4 and Table 2).

**Table 2:** Model fit indices for confirmatory factor analysis.

Factor model	RMSR	RMSEA	GFI	NFI	CFI
Proposed	0.075	0.0.94	0.774	0.720	0.740
Re-specified	0.035^**^	0.046^**^	0.946^**^	0.919^**^	0.967^**^

^*^Indicated acceptable model fit;

^**^Indicated excellent model fit;

RMSR, standardized root mean square residual; RMSEA, root mean square error of approximation; GFI, goodness of fit index; NFI, normed fit index; CFI, comparative fit index.

### 5.2 Internal consistency

The results indicated a good overall consistency of the scale. Excellent internal consistency was achieved within the component “PA recommendation”. The component “personal PA” had an acceptable reliability, while “social support” and “environment” had acceptable internal consistencies (Table 3). Correlations among the four factors were moderate (Table 4). Item-item correlations within each awareness component were all also acceptable (see Tables 5A–D).

**Table 3:** Internal consistencies of the scale and factors.

Factor	Cronbach's alpha	Number of items
Personal PA	0.823^**^	3
Social support	0.741^**^	4
Environment	0.799^**^	7
PA recommendation	0.919^**^	3
Entire Scale	0.857^**^	17

^**^Indicated significant correlations at 0.01 level.

**Table 4:** Factor correlations.

Item	Personal PA	Social support	Environment	Recommendation knowledge
Personal PA	1			
Social support	0.166^**^	1		
Environment	0.299^**^	0.330^**^	1	
PA recommendation	0.322^**^	0.313^**^	0.497^**^	1

^**^Indicated significant correlations at 0.01 level.

**Table 5A:** Item-item correlations within personal PA awareness.

Item	1	2	3
1. During a typical week, I know how much moderate physical activity I have performed.	1		
2. During a typical week, I know how much vigorous physical activity I have performed.	0.630^**^	1	
3. During a typical week, I know how much musclestrengthening physical activity I have performed.	0.522^**^	0.688^**^	1

^**^Indicated significant correlations at 0.01 level.

**Table 5B:** Item-item correlations within social support awareness.

Item	1	2	3	4
1. I know how to find needed supported from my family	1			
2. I know how to find needed supported from friends	0.534^**^	1		
3. I know how to find needed supported from my peers	0.383^**^	0.601^**^	1	
4. I know how to find needed supported from my professors	0.263^**^	0.252^**^	0.478^**^	1

^**^Indicated significant correlations at 0.01 level.

**Table 5C:** Item-item correlations within PA recommendation awareness.

Item	1	2	3
1. I know about physical activity recommendations for young adults on daily total physical activity time.	1		
2. I know about physical activity recommendations for young adults on daily total moderate and vigorous physical activity time.	0.838^**^	1	
3. I know about physical activity recommendations for young adults on frequency of muscle strengthening physical activity per week.	0.738^**^	0.804^**^	1

^**^Indicated significant correlations at 0.01 level.

**Table 5D:** Item-item correlations within environment awareness.

Item	1	2	3	4	5	6	7
1. I know a great deal about the indoor facilities.	1						
2. I know a great deal about the outdoor facilities.	0.430^**^	1					
3. I know a great deal about the accessible stairways.	0.269^**^	0.288^**^	1				
4. I know a great deal about the physical activity courses.	0.451^**^	0.383^**^	0.278^**^	1			
5. I know a great deal about the conceptual physical education courses.	0.368^**^	0.365^**^	0.374^**^	0.594^**^	1		
6. I know a great deal about the group exercise classes.	0.364^**^	0.347^**^	0.236^**^	0.459^**^	0.461^**^	1	
7. I know a great deal about the sports clubs.	0.285^**^	0.369^**^	0.204^**^	0.336^**^	0.410^**^	0.407^**^	1

^**^Indicated significant correlations at 0.01 level.

### 5.3 Additional evidence of construct validity

To further evaluate the psychometric properties of the PA awareness scale, composite reliability (CR), average variance extracted (AVE), and discriminant validity were examined. The results indicated that CR values for all constructs ranged from 0.73 to 0.90, exceeding the recommended threshold of 0.70, indicating satisfactory internal consistency at the construct level. The AVE values were 0.45 for Personal PA awareness, 0.29 for Social Support awareness, 0.23 for Environmental awareness, and 0.68 for Recommendation Knowledge. Although AVE values for three constructs were slightly below the recommended threshold of 0.50, all constructs demonstrated adequate composite reliability, supporting acceptable convergent validity at the construct level. Discriminant validity was assessed using the Fornell–Larcker criterion. The square root of AVE for each construct was greater than or comparable to its correlations with other constructs, indicating that each dimension of PA awareness is empirically distinct while remaining theoretically related. With the exception of a marginal overlap between Environmental awareness and Recommendation Knowledge (√AVE = 0.48 vs. *r* = 0.497), all constructs satisfied the criterion. Overall, these findings provide additional evidence supporting the reliability and construct validity of the proposed four-factor model.

## 6 Discussion

Physical activity (PA) awareness represents a critical yet underexplored psychological mechanism underlying PA behavior. The lack of theoretically sound, valid and reliable instrument for measuring PA awareness has limited the potential to study the psychological effects on the comprehensive behavior change in PA research. The present study contributes to the literature by conceptualizing PA awareness as a multidimensional construct and developing a psychometrically sound instrument measuring PA awareness among college students. It followed a qualitative study that explored how college student perceived their PA experience in relation to their mixed environment, which informed the generation of a large pool of items. The study included scale development and scale validation. The findings support a four-factor structure, including personal PA awareness, PA recommendation awareness, social support awareness, and environmental awareness. Importantly, this structure reflects a theoretically grounded integration of self-awareness theory and the social ecological model. A key theoretical contribution of this study is the explicit mapping between the empirically derived four-factor structure and the underlying theoretical frameworks. The results from CFA affirmed the shared PA experiences from Study I in that college students' PA awareness came from two main dimensions (i.e. internal and external), consisting of four components: personal PA, social support, environmental, and knowledge. A key theoretical contribution of this study lies in clarifying the relationship between the identified four-factor structure and the underlying theoretical frameworks. Specifically, personal PA awareness and PA recommendation awareness correspond to internal evaluative processes emphasized in self-awareness theory, reflecting how individuals monitor and compare their behavior against internalized standards. In contrast, social support awareness and environmental awareness align with the external influences described in the social ecological model, capturing individuals' sensitivity to social and environmental contexts. This mapping demonstrates that PA awareness is not merely a cognitive or knowledge-based construct, but a multidimensional process integrating self-regulation and contextual perception.

By bridging these theoretical perspectives, the study extends existing models of health behavior and provides a more comprehensive understanding of how awareness operates in influencing PA behavior. In addition, the assessment of composite reliability (CR), average variance extracted (AVE), and discriminant validity further strengthened the psychometric evaluation of the scale. The CR values for all constructs exceeded the recommended threshold of 0.70, ranging from 0.73 to 0.90, indicating satisfactory construct reliability. Although the AVE values for social support awareness and environmental awareness were below the conventional cutoff of 0.50, this is not uncommon in constructs involving complex and multidimensional psychological or contextual perceptions. Given that CR values were all adequate, the convergent validity of these constructs can still be considered acceptable at the construct level (54). The observed discriminant validity supported the theoretical distinction among the four dimensions, while also indicating meaningful interrelationships among internal and external aspects of PA awareness. This is consistent with the integrated framework of self-awareness theory and the social ecological model, in which cognitive self-regulation and contextual perception are expected to interact rather than operate independently. The participant-item ration was over 50:1, surpassing the recommended ratio of 10:1, making a sufficient sample size to generate meaningful statistical power (22). Given the lack of existing scales that measure the complex aspects of PA awareness, the study made a remarkable contribution to the body of literature on this topic.

### 6.1 Development of the PA Awareness Scale

The instrument was developed through a number of steps, using previous interview data from college students for item generation, expert reviews for the content validity, a small sample size of college students for the pilot study, and a large sample size of college students for construct validity and reliability. The participants were appropriate for the purpose of the study as they represented the target population.

### 6.2 Sampling issues and response rate

Response rate is an essential parameter to evaluate the effectiveness of data collection in research studies. A high response rate is critical to increase the validity of the results and generalize the findings (24). However, it is often difficult to recruit participants in research. Therefore, a number of different approaches and survey formats were employed to collect sufficient data. The engagement of faculty member and graduate assistants helped greatly in recruiting a fair number of undergraduate students in their classes to participate in the study. Despite that researchers suggested the use of incentives as an effective way to increase response rate (25–27), there were no observations of increased response rates in online survey where extra credits were offered by the instructors in the current study. Unfortunately, it was unclear why the incentives did not work.

Given the mixed types and procedures applied in distributing the survey, it was not feasible to calculate the exact response rate in this study. However, it was shown that paperbased survey had much higher response rate than the online version, as most papers were returned at the time of on-site data collection, which mostly took place in their classroom with instructors' permission. It is interesting to note that even though a paper with QR code printed was provided to the students for convenient access to the survey link, the smartphone version was not used much by the students. Given the extremely low response rate for online survey, the primary researcher visited various campuses to distribute the paper-based survey, which consisted the majority of the responses (*N* = 750).

Although it is common that online survey has lower response rate (i.e. 11% lower) than other methods (24, 25), it should be note that with improvement in technology, the online survey has a number of advantages over paper-based administration, including low cost, flexibility to administer, ease of managing the data, and security for data collecting and storing (28). Most importantly, using online survey may minimize the cases of missing data by changing the setting of a question so that the participants have to complete each question to move forward. However, it is also possible to result in increased drop-out rate as “forcing” the participants to answer every single question may lead to a feeling of frustration. Even though online surveys raised concerns such as difficulty to have a representative sample of the general public due to varied access to the internet (24, 29), it was not a problem in this study, as all students were enrolled undergraduate students with constant internet access.

### 6.3 Survey length

Survey length is beneficial in increasing the reliability of the instrument, however, with an increasing chance of participants' inattentiveness along the time (30). Huang and colleagues (31) defined a term “Insufficient Effort Responding (IER)” as a specific response set in which responder responds to survey measures with low or little motivation to comply with survey instructions, interpret item contents, or to provide accurate responses (31, p. 100). Previous research indicated that web-based surveys have a higher prevalence of IER than paper-based surveys (30, 32, 33), which partially explained the lower response rate of online survey in this study It is suggested to decrease survey length to prevent the occurrence of IER in conducting survey research.

Although the PA awareness scale takes approximately 10–15 min to complete, there were additional questions in the survey following the PA awareness scale for the purpose of Study III, which focused on other related measures such as the 7-day PA level recall using the IPAQ short form, four multiple choice questions that tested real PA recommendation knowledge, and demographic information (i.e. age, gender, ethnicity, height and weight, class standing, major, etc.). Therefore, it might have taken longer than 15 min for the participants to finish the entire survey, especially for the participants that were recruited randomly across different campuses, with the presence of distracting characteristics in the environment. The entire survey practically took longer than estimated (i.e. 20–30 min). However, it was not deemed as a long survey (i.e. 30–45 min), as defined by Galesic and Bsonjak (34).

### 6.4 Construct validity

Construct validity was investigated using CFA. The total number of item was reduced from 30 to 17 to achieve a good model fit. Results of CFA supported the respecified four-factor model, indicating the scale measured four distinct but related constructs. The detailed factor loadings and model fit indices are presented in Table 6. In addition, the final set of items demonstrated face validity (35). Specifically, four factors were identified to delimitate PA awareness in this study: (a) personal PA awareness, defined as the capability of self-evaluating one's own PA behavior; (b) social support awareness, defined as the capability of finding PA support from social relationships; (c) environmental awareness, defined as the knowledge of PA opportunities in the accessible environment; and (d) knowledge awareness, defined as the knowledge of desired PA behaviors. The four-factor model provided empirical support for the theoretically derived structure of the conceptual framework of the proposed conceptual framework, combining self-awareness theory and social ecological model. To further clarify the theoretical positioning of PA awareness, this study explicitly distinguishes it from related constructs frequently examined in physical activity research. First, physical activity knowledge refers to factual understanding of PA guidelines and health benefits, whereas PA awareness involves the perception and evaluation of one's own behavior in relation to such knowledge. Second, self-efficacy reflects confidence in one's ability to perform PA, while awareness does not imply capability or motivation but rather the recognition of one's current behavioral state. Third, while health literacy broadly refers to the capacity to obtain, process, and understand health information to make appropriate health decisions—which can include reflective elements—PA awareness is more narrowly focused on the ongoing, self-referential perception and evaluation of one's own physical activity behavior in relation to both internal standards and external contexts. These distinctions highlight the unique role of PA awareness as a multidimensional construct and suggest that it represents a distinct explanatory mechanism that links cognitive factors and contextual influences to behavioral outcomes.

**Table 6:** Composite reliability, AVE, and discriminant validity of the PA Awareness Scale.

Construct	CR	AVE	√AVE	Personal PA	Social support	Environment	Recommendation knowledge
Personal PA	0.78	0.45	0.67	1			
Social support	0.73	0.29	0.54	0.166	1		
Environment	0.77	0.23	0.48	0.299	0.330	1	
Recommendation knowledge	0.90	0.68	0.82	0.322	0.313	0.497	1

While self-perceived PA levels and PA knowledge have been studied as important indicators of PA awareness in previous studies (36–39), only one factor (i.e. self-reported PA or knowledge) was assessed each time, missing the other three factors as suggested in this instrument. While it is important for an individual to have proper knowledge of PA principles and one's own PA levels, we must also consider the imperative effects of social support and environment on one's PA behavior, in order to provide a more comprehensive description of what should be measured in PA awareness.

The low correlation (*r* = 0.166) between personal PA awareness and social support awareness revealed a high discrimination between these two aspects of PA awareness. Unexpectedly, the correlation between knowledge awareness and environmental awareness was the highest (i.e. *r* = 0.497) among all correlations, given that they were two discrete constructs. The other correlations were moderate (i.e. ranging from 0.299 to 0.330), suggesting moderate discrimination among the subscales. It is reasonable to suggest an underlying cause for the shared variance of these factors (i.e. personal PA, social support and environment) as they were more likely to relate to each other for a regular college student. For example, a student with higher levels of awareness on self-PA is more likely to pay more attention to PA facilities and social support in order to maintain a desirable personal PA level. However, further investigation is needed to explore possible explanations for the correlations among the factors.

### 6.5 Reliability

In the development of the instrument, internal consistencies for the overall scale as well as each factor were assessed. The Cronbach's alpha coefficient for the entire scale was greater than 0.80, indicating acceptable reliability among the sample of college students (55). All between-factor correlations were significantly positive, suggesting that the factors consistently measure of the level of PA awareness. Although relationships between some of the measures in the scale and PA behavior have been observed in previous studies (40–43), to our knowledge, no instruments for assessing PA awareness have been tested for reliability. Therefore, this study contributes to the growth of literature on measurement of PA awareness in college setting.

### 6.6 Reliability for personal PA awareness and recommendation knowledge awareness

The three items of personal PA awareness and three items of PA recommendation awareness demonstrated relatively better internal consistencies (α = 0.823 and α = 0.919, respectively). The items were designed to measure the extend to which students are aware of their own participation levels for each type of PA (i.e. moderate PA, vigorous PA, muscle strengthening PA), as well as the respective PA recommendations. The high consistencies confirmed the importance of an individual's ability of self-monitoring (PA assessment awareness) and goal setting (knowledge awareness) on PA behavior change (44, 45). Self-reported PA, as a most widely used way for self-monitoring, has been used in the vast majority of PA research, however, individuals are prone to systematic self-report bias, resulting in overestimation of desirable behavior (46, 47). It should be noted that the three items in the personal PA awareness subscale aimed to assess the extent to which students were aware of their PA levels rather than their self-estimated amount of PA to avoid erroneous conclusions.

### 6.7 Reliability for external awareness: social support and environment

The internal consistencies for social support and environment awareness were acceptable, although they were slightly lower than the other components (α = 0.741 and 0.799 respectively). Given that these contextual attributes (i.e. social environmental factors) were more dynamic and amenable to change (48, 56), it is reasonable to assume that these items may generate relatively lower consistencies.

Recent studies have explored the measurement of social and environmental factors and their association with PA behavior (40, 42, 48), warranting a brief discussion of measurement issues regarding social environmental factors. PA studies have directly examined the quality of the environment, such as perceived presence or lack of PA resources, and objectively measured quantity of available characteristics (49). No studies have examined the accuracy of such perceptions by participants. In other words, it was not clear to what extend those participants were aware/knowledgeable of their social or physical environment.

In essence, the scale contained an appropriate number of items (i.e. *n* = 17), which only takes about 5 to 10 min. to complete. It is more likely that participants would take the survey when they are asked because it does not take too much time to complete it, increasing the feasibility of using the instrument. More importantly, the instrument supported existing theories in that it consisted of both internal and external awareness highlighted in SAT as well as the 93 multilevel constructs in SEM. As a result, it provided a valid and reliable measurement of PA awareness among college students in the US.

## 7 Limitations

There were a few limitations in Study II. First, the relatively long length of the original survey (i.e., 30 items) may have increased respondent burden, potentially leading to participant fatigue and reducing the likelihood of carefully considered responses for all items. Second, the sampling approach may limit generalizability, as participants were mainly recruited from universities within a single state; therefore, students from other regions and institutional contexts were not represented. Third, given the small sample size of the pilot testing phase, it was insufficient to robustly support full preliminary evaluation of the scale's validity and reliability prior to the main study. Fourth, test–retest reliability was not assessed due to the cross-institutional sampling strategy; nearly half of the participants were recruited randomly from different campuses, making it infeasible to re-contact the same individuals for longitudinal reassessment.

Fifth, and importantly, all data were collected using self-report measures, which may introduce common method bias, including recall bias and social desirability bias. Participants may have overestimated or underestimated their physical activity awareness due to subjective interpretation of items rather than objective assessment. This should be considered when interpreting the strength of the observed associations and factor structure. To partially mitigate this bias, the survey instructions emphasized that there were no correct or incorrect answers and encouraged honest responding; nevertheless, the influence of self-report bias cannot be completely ruled out.

Despite these limitations, several strengths should be noted. First, the sample size was relatively large (*N* = 994), providing sufficient statistical power for exploratory and confirmatory factor analyses. Second, the findings empirically supported the hypothesis that college students' physical activity awareness is a multidimensional construct that can be meaningfully operationalized. Third, the four-factor structure demonstrated acceptable reliability and validity indices, indicating good internal consistency and structural stability. Overall, the final instrument provides preliminary but robust evidence for the proposed dimensionality of physical activity awareness among college students. Future research should aim to replicate this factor structure in diverse samples and consider incorporating objective or device-based measures of physical activity to complement the self-report awareness scale.

## 8 Conclusions and implications

In summary, a number of PA awareness variables were included in the instrument, including personal PA assessment, social support, physical environment and recommendation knowledge, which were key venues for PA promotion (36–39, 50). Unlike previous approaches that focused on single indicators such as self-reported PA or knowledge, the present scale captures the integration of internal self-evaluation and external contextual awareness. Theoretically, this study advances the conceptualization of PA awareness by explicitly integrating self-awareness theory and the social ecological model into a unified framework. Furthermore, by empirically validating the correspondence between the four-factor structure and these theoretical frameworks, the study provides stronger conceptual clarity regarding how PA awareness operates as a multidimensional construct. Methodologically, it provides a reliable and valid tool for measuring this construct. Practically, the findings suggest that enhancing PA awareness across multiple domains may represent a critical pathway for promoting sustained physical activity behavior. Future research should further examine the role of PA awareness as a mediator between knowledge, environmental factors, and behavioral outcomes (51), as well as test the applicability of the scale across diverse populations and contexts.

## Data Availability

The original contributions presented in the study are included in the article/supplementary material, further inquiries can be directed to the corresponding author.
